# Supplementary material for: Time-series transcriptome analysis identified differentially expressed genes in broiler chicken infected with mixed Eimeria species
Source: Front Genet. 2022 Aug 8;13:886781. doi: 10.3389/fgene.2022.886781 (PMC9393255; doi:10.3389/fgene.2022.886781)
Supplement: Supplementary file 2 [file DataSheet1.ZIP › 7dpi_KEGG.Gsea.1625115961950/KEGG_T_CELL_RECEPTOR_SIGNALING_PATHWAY.html]

Details for gene set KEGG\_T\_CELL\_RECEPTOR\_SIGNALING\_PATHWAY[GSEA]

|  || Dataset | TMM\_7dpi\_gct\_format\_7dpi\_gct\_format.Class\_4dpi.cls #PC\_versus\_NC.Class\_4dpi.cls #PC\_versus\_NC\_repos |
| Phenotype | Class\_4dpi.cls#PC\_versus\_NC\_repos |
| Upregulated in class | 0 |
| GeneSet | KEGG\_T\_CELL\_RECEPTOR\_SIGNALING\_PATHWAY |
| Enrichment Score (ES) | -0.47951263 |
| Normalized Enrichment Score (NES) | -1.7011107 |
| Nominal p-value | 0.0 |
| FDR q-value | 0.06892796 |
| FWER p-Value | 0.2748 |
Table: GSEA Results Summary

  

Fig 1: Enrichment plot: KEGG\_T\_CELL\_RECEPTOR\_SIGNALING\_PATHWAY      
 Profile of the Running ES Score & Positions of GeneSet Members on the Rank Ordered List

  

| SYMBOL | TITLE | RANK IN GENE LIST | RANK METRIC SCORE | RUNNING ES | CORE ENRICHMENT || 1 | NFKBIB | na | 369 | 0.859 | -0.0121 | No |
| 2 | MAPK11 | na | 678 | 0.646 | -0.0238 | No |
| 3 | PAK4 | na | 918 | 0.550 | -0.0317 | No |
| 4 | CHP1 | na | 1292 | 0.430 | -0.0536 | No |
| 5 | HRAS | na | 1342 | 0.420 | -0.0485 | No |
| 6 | VAV3 | na | 1390 | 0.408 | -0.0435 | No |
| 7 | CD40LG | na | 1613 | 0.353 | -0.0543 | No |
| 8 | CD4 | na | 2019 | 0.268 | -0.0824 | No |
| 9 | NFKBIA | na | 2532 | 0.187 | -0.1212 | No |
| 10 | PAK5 | na | 2999 | 0.126 | -0.1575 | No |
| 11 | PPP3R1 | na | 3345 | 0.081 | -0.1847 | No |
| 12 | MAP2K2 | na | 3435 | 0.070 | -0.1906 | No |
| 13 | RHOA | na | 3449 | 0.068 | -0.1902 | No |
| 14 | ICOS | na | 3708 | 0.039 | -0.2110 | No |
| 15 | PDCD1 | na | 4153 | -0.012 | -0.2479 | No |
| 16 | JUN | na | 4404 | -0.040 | -0.2680 | No |
| 17 | PPP3CA | na | 4410 | -0.040 | -0.2675 | No |
| 18 | RAF1 | na | 5142 | -0.116 | -0.3263 | No |
| 19 | MAP2K1 | na | 5188 | -0.120 | -0.3274 | No |
| 20 | MAPK13 | na | 5231 | -0.124 | -0.3282 | No |
| 21 | FOS | na | 5340 | -0.134 | -0.3343 | No |
| 22 | CDC42 | na | 5837 | -0.184 | -0.3718 | No |
| 23 | CD28 | na | 5891 | -0.190 | -0.3721 | No |
| 24 | BCL10 | na | 6214 | -0.226 | -0.3942 | No |
| 25 | CTLA4 | na | 6226 | -0.227 | -0.3901 | No |
| 26 | PLCG1 | na | 6241 | -0.228 | -0.3863 | No |
| 27 | NFKBIE | na | 6274 | -0.232 | -0.3839 | No |
| 28 | RELA | na | 6283 | -0.233 | -0.3794 | No |
| 29 | GRB2 | na | 6686 | -0.274 | -0.4071 | No |
| 30 | AKT3 | na | 7033 | -0.312 | -0.4293 | No |
| 31 | PAK2 | na | 7158 | -0.325 | -0.4325 | No |
| 32 | KRAS | na | 7206 | -0.330 | -0.4292 | No |
| 33 | NRAS | na | 7474 | -0.358 | -0.4438 | No |
| 34 | MAPK12 | na | 7612 | -0.373 | -0.4471 | No |
| 35 | MAPK9 | na | 7648 | -0.377 | -0.4417 | No |
| 36 | MAP3K7 | na | 7867 | -0.403 | -0.4512 | No |
| 37 | PDPK1 | na | 8107 | -0.435 | -0.4617 | No |
| 38 | NCK1 | na | 8321 | -0.461 | -0.4694 | Yes |
| 39 | NFAT5 | na | 8371 | -0.468 | -0.4633 | Yes |
| 40 | AKT1 | na | 8399 | -0.472 | -0.4552 | Yes |
| 41 | NFATC1 | na | 8451 | -0.478 | -0.4490 | Yes |
| 42 | IKBKB | na | 8465 | -0.479 | -0.4396 | Yes |
| 43 | PIK3CG | na | 8478 | -0.481 | -0.4300 | Yes |
| 44 | MAPK14 | na | 8502 | -0.484 | -0.4214 | Yes |
| 45 | SOS1 | na | 8869 | -0.533 | -0.4404 | Yes |
| 46 | PAK1 | na | 8943 | -0.541 | -0.4346 | Yes |
| 47 | PPP3CB | na | 8981 | -0.547 | -0.4257 | Yes |
| 48 | NFKB1 | na | 9346 | -0.606 | -0.4430 | Yes |
| 49 | DLG1 | na | 9408 | -0.616 | -0.4346 | Yes |
| 50 | RASGRP1 | na | 9465 | -0.626 | -0.4256 | Yes |
| 51 | CHUK | na | 9554 | -0.639 | -0.4189 | Yes |
| 52 | PIK3R2 | na | 9597 | -0.645 | -0.4083 | Yes |
| 53 | ZAP70 | na | 9604 | -0.646 | -0.3947 | Yes |
| 54 | PTPN6 | na | 9763 | -0.668 | -0.3932 | Yes |
| 55 | CD8A | na | 9773 | -0.669 | -0.3793 | Yes |
| 56 | CARD11 | na | 9981 | -0.708 | -0.3812 | Yes |
| 57 | GRAP2 | na | 10191 | -0.748 | -0.3823 | Yes |
| 58 | CD3D | na | 10250 | -0.758 | -0.3705 | Yes |
| 59 | MAPK3 | na | 10320 | -0.774 | -0.3593 | Yes |
| 60 | PIK3CB | na | 10335 | -0.777 | -0.3435 | Yes |
| 61 | LCP2 | na | 10364 | -0.782 | -0.3287 | Yes |
| 62 | PTPRC | na | 10493 | -0.808 | -0.3217 | Yes |
| 63 | MAP3K14 | na | 10581 | -0.829 | -0.3108 | Yes |
| 64 | LCK | na | 10662 | -0.845 | -0.2990 | Yes |
| 65 | PIK3CD | na | 10707 | -0.855 | -0.2839 | Yes |
| 66 | TEC | na | 10724 | -0.859 | -0.2664 | Yes |
| 67 | PIK3CA | na | 10920 | -0.912 | -0.2628 | Yes |
| 68 | PIK3R5 | na | 10941 | -0.918 | -0.2444 | Yes |
| 69 | CBLB | na | 10996 | -0.935 | -0.2284 | Yes |
| 70 | PIK3R3 | na | 11045 | -0.949 | -0.2116 | Yes |
| 71 | MAP3K8 | na | 11184 | -0.996 | -0.2013 | Yes |
| 72 | CD3E | na | 11250 | -1.017 | -0.1845 | Yes |
| 73 | PIK3R1 | na | 11265 | -1.023 | -0.1632 | Yes |
| 74 | SOS2 | na | 11362 | -1.063 | -0.1480 | Yes |
| 75 | FYN | na | 11389 | -1.076 | -0.1266 | Yes |
| 76 | CD247 | na | 11411 | -1.084 | -0.1046 | Yes |
| 77 | NFATC2 | na | 11531 | -1.128 | -0.0898 | Yes |
| 78 | PAK3 | na | 11679 | -1.225 | -0.0753 | Yes |
| 79 | NFATC3 | na | 11833 | -1.382 | -0.0578 | Yes |
| 80 | CBL | na | 11873 | -1.446 | -0.0294 | Yes |
| 81 | VAV2 | na | 11979 | -1.860 | 0.0026 | Yes |
Table: GSEA details [plain text format]

  

Fig 2: KEGG\_T\_CELL\_RECEPTOR\_SIGNALING\_PATHWAY      
 Blue-Pink O' Gram in the Space of the Analyzed GeneSet

  

Fig 3: KEGG\_T\_CELL\_RECEPTOR\_SIGNALING\_PATHWAY: Random ES distribution      
 Gene set null distribution of ES for **KEGG\_T\_CELL\_RECEPTOR\_SIGNALING\_PATHWAY**
